# Muscle cell derived angiopoietin-1 contributes to both myogenesis and angiogenesis in the ischemic environment

**DOI:** 10.3389/fphys.2015.00161

**Published:** 2015-05-19

**Authors:** Joseph M. McClung, Jessica L. Reinardy, Sarah B. Mueller, Timothy J. McCord, Christopher D. Kontos, David A. Brown, Sabah N. A. Hussain, Cameron A. Schmidt, Terence E. Ryan, Tom D. Green

**Affiliations:** ^1^Department of Physiology, Brody School of Medicine at East Carolina UniversityGreenville, NC, USA; ^2^Diabetes and Obesity Institute, Brody School of Medicine at East Carolina UniversityGreenville, NC, USA; ^3^Department of Pharmacology and Cancer Biology, Duke University School of MedicineDurham, NC, USA; ^4^Medical Scientist Training Program, Duke University School of MedicineDurham, NC, USA; ^5^Division of Cardiology, Department of Medicine, Duke University Medical CenterDurham, NC, USA; ^6^Meakins-Christie Laboratories, Department of Medicine, McGill University Health Centre, Royal Victoria Hospital, McGill UniversityMontreal, QC, Canada; ^7^Department of Critical Care, McGill University Health Centre, Royal Victoria Hospital, McGill UniversityMontreal, QC, Canada

**Keywords:** paracrine, angiogenesis, regeneration, muscle, progenitor cell, ischemia, vascular disease

## Abstract

Recent strategies to treat peripheral arterial disease (PAD) have focused on stem cell based therapies, which are believed to result in local secretion of vascular growth factors. Little is known, however, about the role of ischemic endogenous cells in this context. We hypothesized that ischemic muscle cells (MC) are capable of secreting growth factors that act as potent effectors of the local cellular regenerative environment. Both muscle and endothelial cells (ECs) were subjected to experimental ischemia, and conditioned medium (CM) from each was collected and analyzed to assess myogenic and/or angiogenic potential. In muscle progenitors, mRNA expression of VEGF and its cognate receptors (Nrp1, Flt, Flk) was present and decreased during myotube formation *in vitro*, and EC CM or VEGF increased myoblast proliferation. Angiopoietin-1 (Ang-1), Tie1, and Tie2 mRNA increased during MC differentiation *in vitro*. Exogenous Ang-1 enhanced myogenic (MyoD and Myogenin) mRNA in differentiating myoblasts and increased myosin heavy chain protein. Myotube formation was enhanced by MC CM and inhibited by EC CM. Ang-1 protein was present in CM from MCs isolated from both the genetically ischemia-susceptible BALB/c and ischemia-resistant C57BL/6 mouse strains, and chimeric Tie2 receptor trapping *in situ* ablated Ang-1's myogenic effects *in vitro*. Ang-1 or MC CM enhanced myotube formation in a mixed isolate of muscle progenitors as well as a myoblast co-culture with pluripotent mesenchymal cells (10T1/2) and this effect was abrogated by viral expression of the extracellular domain of Tie2 (AdsTie2). Furthermore, mesh/tube formation by HUVECs was enhanced by Ang-1 or MC CM and abrogated by Tie2 chimeric receptor trapping. Our results demonstrate the ability of muscle and endothelial cell-derived vascular growth factors, particularly Ang-1, to serve as multi-functional stimuli regulating crosstalk between blood vessels and muscle cells during regeneration from ischemic myopathy.

## Introduction

Peripheral artery disease (PAD) is caused by atherosclerosis of the peripheral arteries, most commonly in the lower extremities, and is nearly as prevalent as coronary artery disease (CAD) (Hirsch et al., 2006). Traditionally, research on the limb tissue response to ischemia has focused on angiogenesis and vascular remodeling. In addition to delivery of exogenous vascular growth factors, therapeutic angiogenesis by targeted delivery of transduced myoblasts has been suggested as a promising option for ischemic muscle diseases (Von Degenfeld et al., 2003), and clinical research has recently emphasized the development of stem or progenitor cell therapies to stimulate angiogenesis in patients with PAD (Prather et al., 2009; Fadini et al., 2010, 2012; Lawall et al., 2011; Blum et al., 2012; Volz et al., 2012). In addition to stimulating vascular growth through actions on endothelial cells (ECs), it has been suggested that these approaches may act directly on muscle progenitor cells (Deasy et al., 2009). Despite the identification of skeletal muscle myopathy in the limb muscle of PAD patients (Brass and Hiatt, 2000; Brass et al., 2000, 2004; Pipinos et al., 2007, 2008), skeletal muscle is often not considered as a component of PAD pathology. In fact, the presence of muscle degenerative and regenerative phenotypes in both mouse hindlimb ischemia (HLI) and human PAD are often overlooked or minimized (Yang et al., 2008).

Skeletal muscle possesses an inherent plasticity that allows it to recover from substantial insult. This plasticity involves the coordination of processes involved in vascular growth (angiogenesis) and maintenance, extracellular matrix deposition, and nerve and muscle fiber regeneration (Charge and Rudnicki, 2004; Gehrig and Lynch, 2011; Winkler et al., 2011). Because measures of muscle strength/integrity have been correlated with mortality in patients with PAD (McDermott et al., 2012), enhanced muscle regeneration in response to ischemia could represent a potential critical source of pro-angiogenic factors in the local ischemic limb environment. The mechanisms of cross-talk between skeletal muscle and endothelial cells during periods of injury and stress remain poorly defined, but may be representative of complex and novel evolutionarily conserved mechanisms for overall tissue survival and recovery from pathological insults. Despite differences in the transcriptional responses of the various cell types in regenerating skeletal muscle tissue (Arany et al., 2008), increasing evidence demonstrates that their responses to injury are critically linked through regulatory factors traditionally thought to target blood vessels exclusively. Among the early genes expressed in response to skeletal muscle injury are several angiogenic growth factors, including vascular endothelial growth factor (VEGF) and the angiopoietins (Ang-1, Ang-2), and their cognate receptors (Wagatsuma, 2007). Like VEGF, the effects of the angiopoietins are not specific for vascular endothelial cells, as their receptors (Tie1, Tie2) are known to be expressed in hematopoietic cells and they have also recently been shown to be expressed in skeletal muscle cells (Chazaud et al., 2003; Abou-Khalil et al., 2009).

The close proximity of local muscle progenitor cell populations, including both satellite cells and pericytes, to resident endothelial cells (Abou-Khalil et al., 2010) may facilitate unique responses to the ischemic and myopathic environment that exists in PAD. For example, VEGF, which is upregulated by cellular hypoxia, is known to drive angiogenesis as well as stimulate muscle regeneration (Germani et al., 2003; Arsic et al., 2004; Messina et al., 2007). Accordingly, Rissanen et al. (2002) have immunohistochemically verified that atrophic and regenerating myofibers in patients with both acute and chronic lower limb ischemia express not only VEGF but also VEGF receptors. We recently demonstrated that another angiogenic factor, Ang-1, improves muscle contractility, myofiber regeneration, and capillary density in response to toxin-mediated muscle injury in mice (Mofarrahi et al., 2015). Furthermore, we co-localized Ang-1 expression to satellite cells in the regenerating environment and verified a myogenic role for Ang-1 in myoblast fusion. These findings suggest a novel dual role for traditional vascular growth factors as potential myogenic regulatory factors in limb ischemia. The local production of these growth factors and how they modulate both myogenesis and angiogenesis in the ischemic limb are not well understood, but could potentially lead to the development of novel therapies targeting the skeletal myopathy associated with critical limb ischemia (CLI). To explore this idea, we tested the hypothesis that vascular growth factors secreted by endothelial and muscle cells during experimental ischemia affect both myogenesis and angiogenesis by acting in both a paracrine and autocrine fashion to alter muscle progenitor and endothelial cell biology.

## Materials and methods

### Animals

Experiments were conducted on adult C57BL/6 and BALB/c mice and approved by either East Carolina University or Duke University Medical Center Institutional Animal Care and Use Committees. All animal care complied with the *Guide for the Care and Use of Laboratory Animals*, Institute of Laboratory Animal Resources, Commission on Life Sciences, National Research Council. Washington: National Academy Press, 1996.

### RNA isolation and RT-PCR

Total RNA was extracted using Trizol-phenol/chloroform and reverse-transcribed using Superscript III Reverse Transcriptase Kit and random hexamer primers (Invitrogen Inc.). Real-time PCR was performed using a 7500 Real-Time PCR System (Applied Biosystems, Foster City, CA). Relative quantification of mRNA levels was determined using the comparative threshold cycle (Δ Δ CT) method using FAM TaqMan® Gene Expression Assays (Applied Biosystems) specific to each gene run in complex (multiplex) with a VIC labeled GAPDH control primer.

### Primary antibodies and materials

The following commercial antibodies were used: MyHC (MF20, Developmental Studies Hybridoma Bank, University of Iowa), α-tubulin (Sigma-Aldrich), DAPI mounting medium (VECTOR Laboratories, H-1200) and Ang-1 (AF923, R&D Systems). Recombinant mouse Tie-2 Fc Chimera (762-T2-100; R&D Systems), recombinant human Ang-1 (500 ng/mL; R&D Systems), recombinant human Ang-2 (500 ng/mL; R&D Systems), recombinant human VEGF (50 ng/mL; R&D Systems), human basic fibroblast growth factor (5 ng/mL; R&D Systems), and recombinant basic hepatocyte growth factor (500 ng/mL; R&D Systems) were used in cell culture experiments.

### Immunoblotting

SDS-PAGE and WB were performed according to standard methods. Blots were visualized with chemiluminescence using standard film procedures. Blotting for Ang-1, specifically, was performed as previously detailed (Davis et al., 1996). Loading and transfer of equal amounts of protein was confirmed by Ponceau staining and probing with anti-α-tubulin.

### Cell lines and culture

Primary murine muscle precursor cells (mouse myoblasts) were prepared as previously described (Rosenblatt et al., 1995; McClung et al., 2012). Briefly, muscle cells (MCs) were isolated from 6-week-old C57BL/6J or BALB/cJ mice, preplated for purification, and grown in growth medium (Ham's F10 supplemented with 20% FBS, Pen-Strep (P/S/A) and 5 ng/mL rbasic FGF for 6 days. DMEM supplemented with 10% FBS and P/S/A was used to maintain immortalized murine C2C12 cells, which were purchased from ATCC. C3H-10T1/2 pluripotent cells were purchased from ATCC and maintained in Eagle's Basal medium with 2 mM L-glutamine, 1.5 g/L sodium bicarbonate and Earle's BSS supplemented with 10% heat-inactivated FBS and P/S/A. Previously described (Misteli et al., 2010) LacZ positive primary myoblasts from C57BL/6 mice were also utilized for gene expression and fusion experiments. Reduced serum medium (DMEM supplemented with 2% HoS, P/S/A, and.1% Insulin/Selenium/Transferrin) was utilized to induce myotube formation/differentiation. Briefly, cells were rinsed twice in 1X phosphate-buffered saline (PBS) and returned to standard culture conditions in differentiation medium (DM) for the indicated times, and medium was changed every 24-h. Human umbilical vein endothelial cells (HUVECs) were isolated as previously described from donor placental umbilical veins (Harfouche et al., 2003). To evaluate the effects of ischemia/hypoxia in skeletal muscle and (ECs) *in vitro*, we have established a model of cellular hypoxia in which cells are subjected to 0.0% O_2_ and deprived of nutrients in Hanks' balanced salt solution (HBSS) (Arany et al., 2008) to mimic the local environment resulting from severe ischemia in PAD (referred to hereafter as hypoxia+nutrient deprivation, HND). HND was performed to generate conditioned medium (CM) from muscle myotubes or ECs. Endothelial cell proliferation was assessed using a hemocytometer and standard culture techniques. Muscle myoblast cell proliferation was assessed by methanol fixation, staining with hematoxylin, and image analysis.

### Fusion index and EC cord/tube formation assay

For fusion index analysis, myotubes were washed with PBS, fixed with 100% methanol for 5 min, left to air dry for 10 min, permeabilized in 0.25% Triton X-100/PBS, immunofluorescently labeled with MF-20 (Myosin) antibody overnight at 4°C followed by 2 rinses in PBS. Cells were then incubated with IgG2b Alexa Fluor 488 secondary antibody for 1 h at RT, rinsed twice in PBS, and mounted in VECTASHIELD DAPI mounting medium. All wells within a single experiment were labeled simultaneously using the same batch of antibody dilutions and solutions. Each well was photographed in five randomly selected regions using an inverted microscope camera system. The number of myonuclei and the total number of nuclei were scored and the fusion index was calculated as the percentage of total nuclei incorporated in myotubes, which were defined as having > 2 nuclei. To verify the effects of exogenous Ang-1 we used an adenoviral vector encoding soluble Tie2 (AdsTie2) (Lin et al., 1998). Co-culture experiments were performed by plating C3H-10T1/2 pluripotent cells at a 75–25% ratio with C2C12 myoblasts in 6-well plates coated with entactin, collagen, laminin (ECL, 08-110 Millipore) and allowed to adhere and reach 50–60% confluence in primary GM overnight. For the analysis of the effects of angiopoietin-1 scavenging on co-culture fusion index, approximately 100,000 cells at a 75/25% ratio of C3H-10T1/2 to C2C12 were plated on 12-well plates coated with ECL and allowed to adhere and reach 50–60% confluence in primary GM overnight, and infected with either control AdGFP, or AdsTie2 for 24 h in DM. DM was then changed every 24 h for 3-days for a total of 96-h of differentiation. To verify the presence and role of Ang-1 in ischemic CM we utilized a chimeric Tie2 receptor trap. Briefly, CM was incubated overnight with fcTie2 at 4°C with rotation. IgG agarose beads were then used to immunoprecipitate associated proteins and Ang-1 depletion was verified by western blotting. Mesh/loop formation in HUVECS was performed by plating approximately 15000 HUVEC cells in each well of a Matrigel (354263, Corning) coated 48-well plate, and incubating for 8-h at 37°C and 5.0% CO_2_. Cells were then rinsed in 1XPBS and fixed for 20 min in 4% Paraformaldehyde before phalloidin/DAPI staining and imaging on an inverted microscope. Each well was photographed in three randomly selected regions and the number of meshes/loops was quantified manually.

### Statistical analysis

Statistical analyses were carried out using StatPlus:mac (v 2009) statistical software. Data were compared using ANOVA and Student's 2-tailed *t*-test. In all cases, *P* < 0.05 was considered statistically significant.

## Results

**Figure 1:**
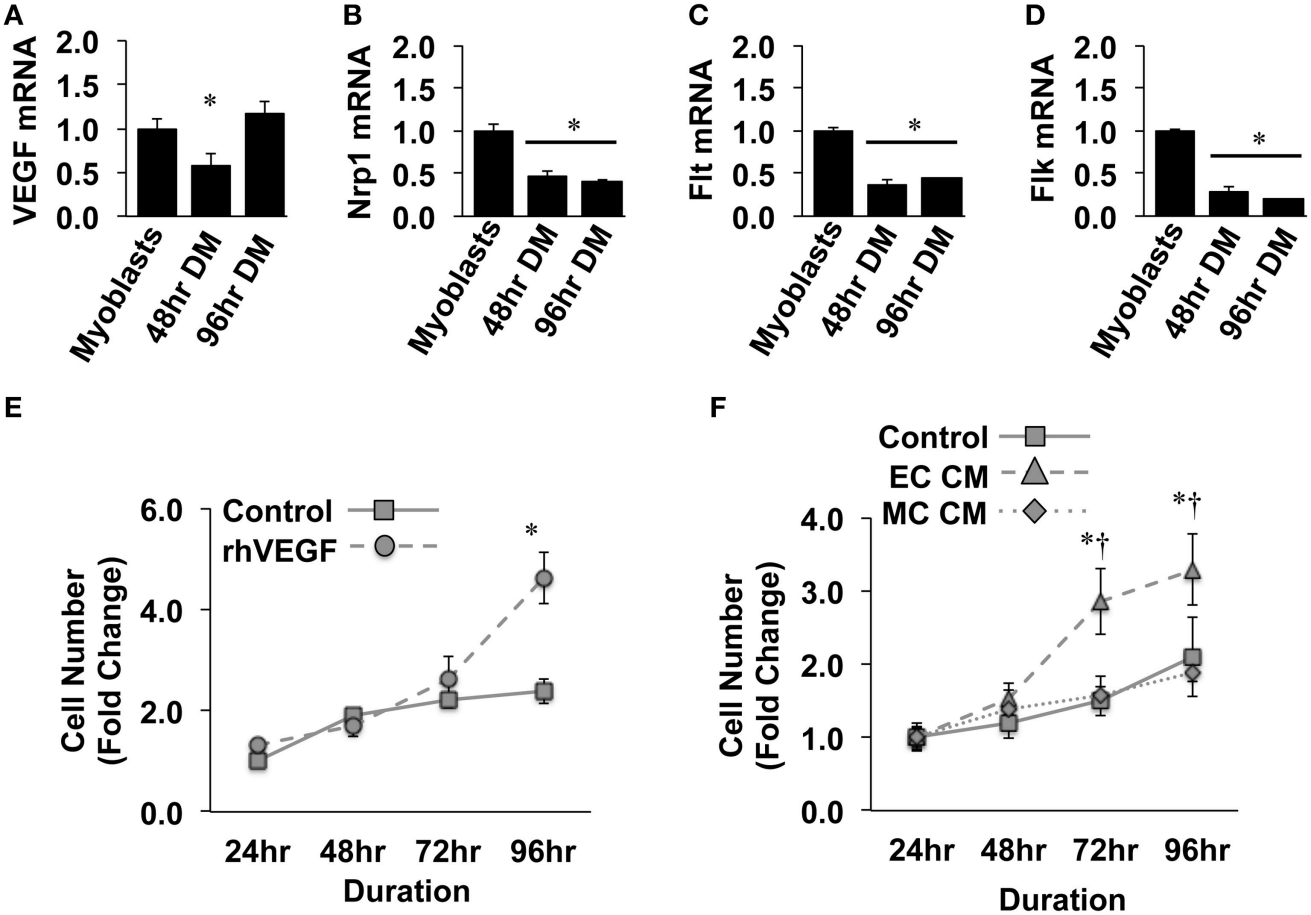
**Ischemic endothelial cells promote muscle precursor cell (MPC) proliferation. (A–D)** Muscle myoblasts were grown to confluence and placed in differentiation medium (DM) for 48- or 96-h. QRT-PCR was used to determine the mRNA expression levels of VEGF **(A)**, Neuropilin/Nrp1 **(B)**, VEGFR1/Flt **(C)**, and VEGFR2/Flk **(D)**. **(E)** Primary mouse MPCs were plated at approximately 20% confluence, treated with recombinant VEGF (50 ng/mL), and cell number was determined at the indicated time points. **(F)** Primary mouse MPCs were plated at approximately 20% confluence, treated with conditioned medium from muscle cells (MC CM) or HUVECs (EC CM) that had been subjected to experimental ischemia (HND), and cell number was determined at the indicated time points. Cell proliferation rates were normalized to Control values at 24-h post-plating. ^*^P < 0.05 vs. Myoblasts (QRT-PCR) or time-matched control (proliferation). ^†^*P* < 0.05 vs. time-matched MC CM treatment group. All values are means ± SE.

Previous reports have detailed specific effects of traditional vascular growth factors on ischemic muscle tissue. VEGF, in particular, has been widely studied in the context of ischemic angiogenesis in skeletal muscle tissue (Rissanen et al., 2001; Iwaguro et al., 2002; Arsic et al., 2004; Yan et al., 2005; Karkkainen et al., 2009; Jazwa et al., 2013), and is known to be produced in regenerating myofibers (Rissanen et al., 2002). For these reasons, we initially verified the expression of VEGF and its cognate receptors (VEGFR1/Flt, VEGFR2/Flk, and Neuropillin/Nrp1) in differentiating muscle progenitor cells. During myotube formation (48 and 96-h), VEGF, Nrp1, Flt, and Flk gene expression decreased compared to that observed in confluent myoblasts (Figures 1A–D). Primary MPC proliferation was increased in the presence of recombinant VEGF (Figure 1E), corroborating previously reported expression patterns and effects of VEGF on MPCs *in vitro* (Germani et al., 2003). The proliferative effect of recombinant VEGF on MPCs was replicated with conditioned medium generated from experimentally ischemic endothelial cells (EC CM), but not muscle cells (MC CM) (Figure 1F), indicating the potential for ischemic endothelial cells to drive myoblast proliferation and subsequent muscle regeneration in the ischemic environment.

**Figure 2:**
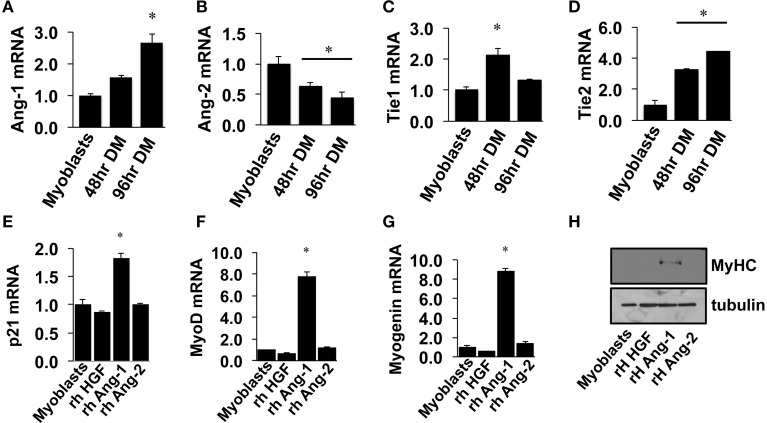
**Angiopoietin-1 enhances myotube formation. (A–D)** Myoblasts were grown to confluence and allowed to differentiate for 48- or 96-h. QRT-PCR was used to determine the mRNA expression levels of Ang-1, Ang-2, Tie1, and Tie2. (**E–G)** Myoblasts were grown to confluence and allowed to differentiate for 24 h with exogenous administration of recombinant hepatocyte growth factor (HGF; 500 ng/mL), Ang-1 (500 ng/mL), or Ang-2 (500 ng/mL). QRT-PCR was used to determine the mRNA expression of the cell cycle regulator p21 **(E)** and the myogenic regulatory factors MyoD **(F)** and Myogenin **(G)**. **(H)** Myoblasts differentiated for 24-h with exogenous HGF, Ang-1, or Ang-2 were analyzed for myosin heavy chain (MyHC) protein. ^*^P < 0.05 vs. Myoblast. All values are means ± SE.

**Figure 3:**
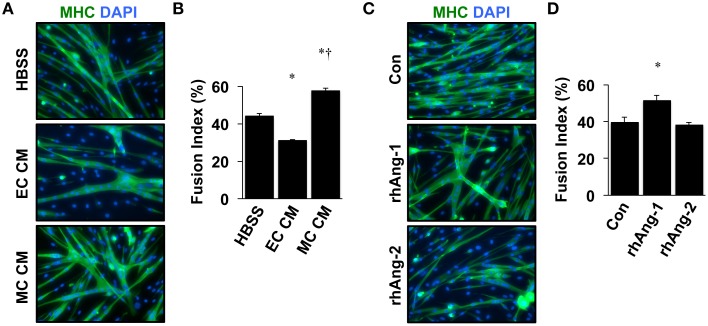
**Ischemic myotubes and Ang-1 promote myoblast differentiation. (A)** Representative immunofluorescently labeled images of differentiated myotubes (96 h) for visualization of myosin heavy chain (MHC, green) and nuclei (DAPI, blue) after treatment in differentiation medium supplemented with 25% by volume vehicle (HBSS) or conditioned medium from experimentally ischemic HUVECs (EC CM) or myotubes (MC CM). **(B)** Quantification of the percentage of nuclei incorporated into multinucleated myotubes (Fusion Index, %) after CM treatment. **(C)** Representative images of immunofluorescently labeled differentiated myotubes (96 h) after treatment in differentiation medium supplemented with Ang-1 (500 ng/mL), or Ang-2 (500 ng/mL) Green: myosin heavy chain (MHC); blue: DAPI. **(D)** Quantification of the percentage of nuclei incorporated into multinucleated myotubes (Fusion Index, %) after angiopoietin treatment. ^*^P < 0.05 vs. Control or Myoblast, ^†^*P* < 0.05 vs. EC CM treatment group. All values are means ± SE.

Similar to VEGF, angiopoietin signaling has recently been recognized for its role as an effector of muscle cells (Abou-Khalil et al., 2009; Lee et al., 2013; Mofarrahi et al., 2015), and its expression in muscle progenitors and ability to enhance muscle regeneration from toxin-induced injury has been shown (Mofarrahi et al., 2015). The potential paracrine influence of Angiopoietins on both muscle and endothelial cells, therefore, makes it a viable target for therapeutic intervention during ischemia as well. We examined the mRNA expression patterns of the angiopoietins (Ang-1 and Ang-2) and their cognate receptors (Tie1 and Tie2) over the timeline of MPC differentiation. Ang-1 mRNA increased with myotube formation (Figure 2A), while Ang-2 mRNA decreased (Figure 2B). Expression of Tie1 and Tie2 mRNA increased during myotube formation (Figures 2C,D). Treatment of confluent myoblasts with exogenous Ang-1 for 24 h increased mRNA expression of differentiation (p21; Figure 2E) and myogenic regulatory factors (MyoD and Myogenin; Figures 2F,G) and the protein expression of myosin heavy chain (Figure 2H). These findings corroborated our previous data from human muscle cells (Mofarrahi et al., 2015) and demonstrate both a coordinated pattern of expression of Angiopoietin and cognate receptor gene expression during myotube formation and the myogenic influence conferred specifically by Ang-1. To determine the ability of ischemic muscle or endothelial cells to express factors that could influence myogenesis, MPCs, and ECs were subjected to HND as a means of inducing experimental ischemia, and effects on myotube formation were tested. Conditioned medium from ischemic endothelial cells (EC CM) inhibited myotube formation, whereas conditioned medium from ischemic myotubes (MC CM) enhanced the fusion of MPCs into multinucleated myotubes (Figures 3A,B). Interestingly, we could re-capitulate this effect by treating differentiating myoblasts daily with exogenous recombinant Ang-1, but not Ang-2 (Figures 3C,D).

**Figure 4:**
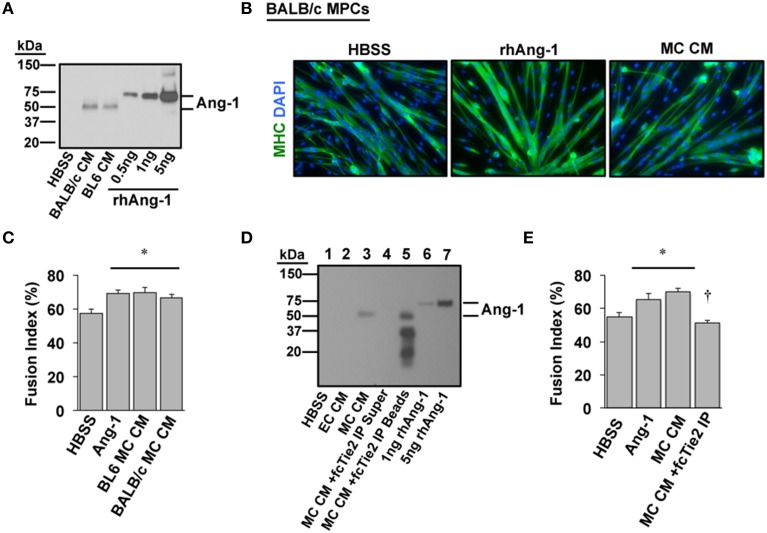
**Angiopoietin-1 is secreted by ischemic myotubes to promote muscle cell differentiation. (A)** Western blotting of CM from primary myotubes derived from ischemia-susceptible (BALB/c) and ischemia-resistant (BL6) parental strains verifies the presence of Ang-1 protein. **(B)** Representative images of primary BALB/c muscle progenitor cells differentiated into myotubes (96 h) after treatment in differentiation medium supplemented with vehicle (HBSS), Ang-1 (500 ng/mL), or conditioned medium from experimentally ischemic BALB/c myotubes (MC CM). Green: myosin heavy chain (MHC); blue: DAPI. **(C)** Quantification of the percentage of nuclei incorporated into multinucleated myotubes (Fusion Index, %) after treatment in differentiation medium supplemented with vehicle (HBSS), Ang-1 (500 ng/mL), or conditioned medium from experimentally ischemic BL6 or BALB/c myotubes (MC CM). **(D)** To verify the presence and role of Ang-1 in ischemic CM we utilized a chimeric Tie2 receptor trap. MC CM was incubated overnight with fcTie2 at 4°C with rotation. IgG agarose beads were then used to immunoprecipitate associated proteins. Ang-1 depletion was verified by western blotting. Representative lanes: ^1^HBSS Vehicle Control; ^2^EC CM; ^3^MC CM; ^4^fcTie2 depleted MC CM Supernatant; ^5^fcTie2 depleted MC CM Bead/protein aggregate; ^6^1 ng recombinant human Ang-1; ^7^10 ng recombinant human Ang-1. **(E)** Effects of depleting Ang-1 from MC CM on myotube formation. ^*^P < 0.05 vs. HBSS Control. ^†^*P* < 0.05 vs. MC CM or Ang-1. All values are means ± SE.

We next sought to verify whether Ang-1 is indeed a component of MC CM and whether its presence is required to confer the myogenic effects of MC CM in primary cell cultures. A mouse strain-dependent regenerative response occurs *in vivo* and *in vitro* with HLI. Specifically, some inbred mouse strains (e.g., BALB/c) display marked tissue necrosis in response to HLI whereas other strains (e.g., C57BL/6) show little to no tissue injury (Dokun et al., 2008). We previously determined that the varied pathological responses among strains include differential expression of the Angiopoietins and their cognate receptors in muscle tissue. Therefore, we also aimed to identify any differences in Ang-1 secretion with ischemia in primary myotube cultures from ischemia-resistant (BL6) or -susceptible (BALB/c) mice. Western blotting revealed that Ang-1 protein was indeed secreted into MC CM but was not differentially expressed by mouse strain (Figure 4A). Treatment of differentiating BALB/c myoblasts with exogenous Ang-1 or MC CM derived from either BL6 or BALB/c myotubes significantly improved myotube formation (Figures 4B,C), suggesting the absence of strain specific deficits in ischemic myotube Ang-1 secretion. Depletion of Ang-1 from MC CM by the addition of recombinant Tie2-Fc protein abrogated the myogenic effects of the MC CM on primary cultures (Figures 4D,E).

**Figure 5:**
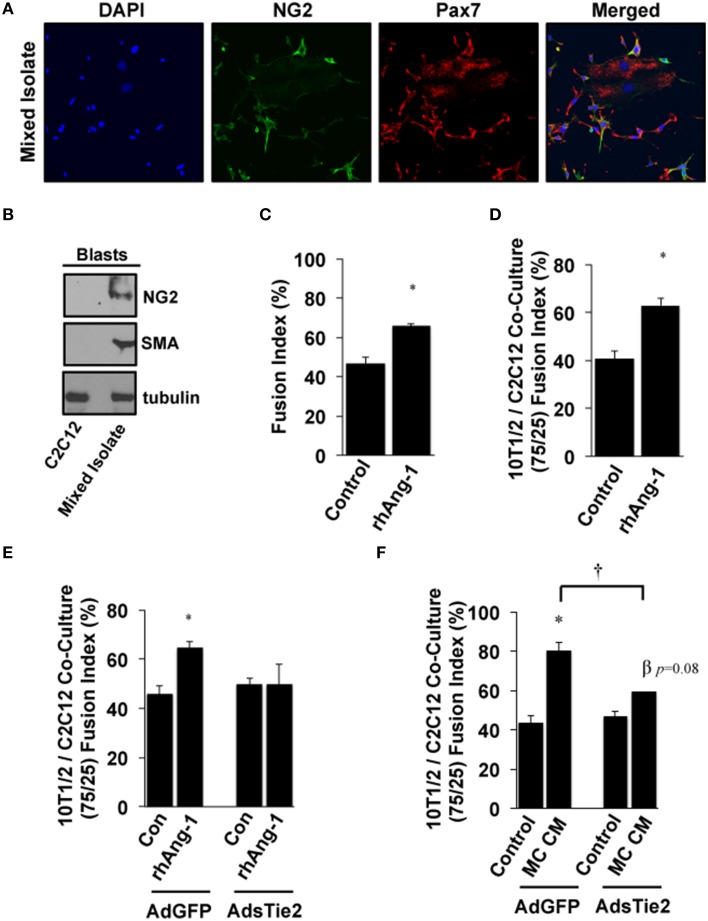
**Effects of Ang-1 on pluripotent cell myotube formation. (A,B)** Mixed primary isolates including peri-endothelial progenitors and MPCs were immunofluorescently labeled **(A)** or western blotted **(B)** to verify NG2 and/or Pax7 expression to demonstrate the relative expression of NG2 and smooth muscle actin (SMA) compared to that in immortalized C2C12 myoblasts. **(C)** Ang-1 effects on mixed cell isolate differentiation into myotubes (Fusion Index, %). **(D)** C3H-10T1/2 pluripotent cells and C2C12 myoblasts were plated at a 75:25 ratio. Cells were allowed to adhere and then treated with Ang-1 and analyzed for myoblast fusion rates after 120 h of low serum culture. **(E,F)** Effects of inhibiting bio-available Ang-1 in MC CM on myotube formation in C3H-10T1/2:C2C12 co-cultures. Co-cultures were infected with either control AdGFP, or AdsTie2 for 24 h and analyzed for myoblast fusion after a total of 120 h of reduced serum culture with Ang-1 or MC CM supplementation. ^*^P < 0.05 vs. Control or virus-matched Control. ^†^*P* < 0.05 vs. AdGFP MC CM. β *P* = 0.08 vs. AdExTEK Control. All values are means ± SE.

Based on these results and the fact that multiple progenitor populations can contribute to muscle regeneration after myopathy (Abou-Khalil et al., 2010), including Pax7^+^ satellite cells and NG2^+^ pericytes, we next examined the myogenic potential of Ang-1 and MC CM in co-cultures of pluripotent cells. Mixed isolates of primary cells were prepared from mouse skeletal muscle. Cellular heterogeneity was verified by immunofluorescence microscopy for Pax7 and NG2 (Figure 5A) and by Western Blotting for NG2 and SMA (Figure 5B). Myotube formation in these mixed isolates was significantly increased by treatment with exogenous Ang-1 (Figure 5C). In parallel experiments, immortalized 10T1/2 mesenchymal stem cells and C2C12 myoblasts were co-cultured at a 75/25% ratio (10T1/2:C2C12) and induced to differentiate. Similar to the mixed primary isolates, myotube formation in these co-cultures was also enhanced by the addition of Ang-1 (Figure 5D). We next verified a requirement for Ang-1 in this process by adenoviral overexpression of the soluble extracellular domain of Tie2 (AdsTie2) to sequester Ang-1 in the culture medium. AdsTie2 attenuated the myogenic potential of both exogenous rhAng-1 (Figure 5E) and MC CM (Figure 5F) in 10T1/2:C2C12 co-cultures. Collectively, these studies demonstrate that the myogenic potential of ischemic MC CM on mixed muscle progenitor cell populations is mediated, at least in part, by Ang-1.

**Figure 6:**
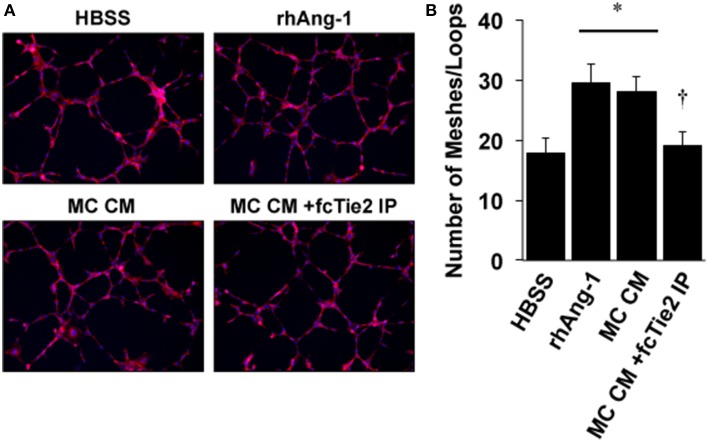
**Conditioned medium from ischemic muscle cells enhances endothelial cell mesh/loop formation. (A)** HUVECs were plated on Matrigel and imaged after 8-h of treatment in medium supplemented with 25% by volume vehicle (HBSS), Ang-1 (500 ng/mL), conditioned medium from experimentally ischemic myotubes (MC CM), or conditioned medium from experimentally ischemic myotubes (MC CM) after Ang-1 depletion with Tie2-Fc. **(B)** Analysis of mesh/loop formation. For six replicate experiments the mean mesh/tube number was calculated from three fields of view. ^*^P < 0.05 vs. HBSS. ^†^ vs. rhAng1 or MC CM. All values are means ± SE.

We next initiated a series of experiments to determine whether ischemic MC CM could confer angiogenic properties on endothelial cells and whether these angiogenic effects were also due to the presence of Ang-1 in the CM. Using a standard angiogenesis assay, we examined the effects of MC CM and Ang-1 depleted MC CM on HUVEC mesh/tube formation *in vitro* (Figure 6A). As expected, tube formation was significantly increased by exogenous Ang-1 treatment, as previously described (Cho et al., 2004). However, MC CM also increased tube formation, and this effect was attenuated by treatment of MC CM with recombinant Tie2-Fc protein (Figure 6B), demonstrating the potential for paracrine effects of ischemic muscle cells on the surrounding vasculature.

## Discussion

In this report we demonstrate that muscle and endothelial cells exposed to experimental ischemia *in vitro* secrete angiogenic factors that are capable of regulating muscle progenitor cell proliferation and muscle progenitor and pluripotent cell differentiation into myotubes, as well as endothelial tube formation. We utilized recombinant VEGF and Ang-1, as well as a soluble Tie2 as an Ang-1 trap to determine that Ang-1 is primarily responsible for the effects of ischemic MC CM on myoblasts, pluripotent progenitor cells, and endothelial cells. These findings provide biological context for our previous observation of the expression of vascular growth factor receptors, including Tie and VEGF receptors, on MPCs^12^ and our work verifying a specific role for Ang-1 in muscle regeneration (Mofarrahi et al., 2015). Moreover, these findings demonstrate that these signaling pathways are a component of the cellular tissue response to ischemic muscle injury that links endogenous progenitors and endothelial cells.

A key component of the genetically directed response of limb tissue to ischemia is the degenerative and regenerative response of the skeletal muscle to the insult. We previously described a rapid and drastic shift of skeletal muscle cells toward catabolic and apoptotic processes when exposed to hypoxia (McClung et al., 2012). These cell types appear individually programmed to respond in ways that preserve vascular integrity while sacrificing muscle cell homeostasis. Intricate coordination of the pathways mediating muscle plasticity may be required to allow tissue survival and facilitate recovery until blood flow can be fully restored by angiogenesis and/or collateral vessel formation. The individual effects of MC CM, EC CM, and vascular growth factors in the current study represent a pattern that stimulates cellular proliferation (VEGF and/or EC CM) and subsequent muscle regeneration and vascular maturation (Ang-1 and/or MC CM). In this regard, inhibition of MC fusion by EC CM suggests that the timing of secreted factors is absolutely critical to efficiently direct the cellular processes enacted by ischemia. Our previous work has also demonstrated the temporal efficiency of Ang-1 to drive muscle regeneration in the absence of a direct ischemic event (Mofarrahi et al., 2015). In fact, exogenous administration of Ang-1 days after the initial injury was still sufficient to enhance muscle recovery and improve capillary density. Dysfunctional timing of the secretion of these factors by ischemic resident cells could inhibit tissue recovery and exacerbate the pathology of ischemia. Our findings demonstrate the plausibility of developing a coordinated and sequential growth factor therapy targeting multiple cellular compartments to enhance regeneration in the myopathic ischemic limb.

Although Ang-1 has been shown to have effects on MPCs (Dallabrida et al., 2005; Wagatsuma, 2007; Abou-Khalil et al., 2009; Volz et al., 2012; Mofarrahi et al., 2015), its effects are still somewhat controversial, and little is known about its effects on muscle regeneration in the context of ischemia. The angiopoietins are a unique family of growth factors (including Ang-1-4) that have context-dependent agonist or antagonist cellular effects despite binding Tie2 with similar affinity, and Ang-1 and -2 have been shown to be critical for the remodeling and maintenance of blood vessels (Fukuhara et al., 2010; Reiss, 2010). Evidence has shown that skeletal muscle cells respond to the angiopoietins both as myoblasts and as differentiated myotubes in a manner previously shown to be at least partly integrin receptor-dependent (Dallabrida et al., 2005). Mature murine myotubes display much greater responsiveness to Ang-1 *in vitro* than do undifferentiated myoblasts (Dallabrida et al., 2005). Although this difference was originally attributed to increased integrin subunit expression with myotube maturation, markedly greater expression of Tie2 with myoblast differentiation may also be responsible for this effect (Abou-Khalil et al., 2009). However, our results suggest that, regardless of whether Ang-1 acts through Tie2 or integrins, exogenous Ang-1 has potential therapeutic utility for skeletal myopathies in addition to its potential for improving vascular growth and stabilization.

Satellite cells reside in close proximity to both myonuclei and capillary endothelial cells, and they are activated and induced to differentiate by various growth factors, including basic fibroblast growth factor (bFGF), insulin-like growth factor-1 (IGF-1), hepatocyte growth factor (HGF), vascular endothelial growth factor (VEGF), and platelet-derived growth factor (PDGF), some of which can be expressed by endothelial cells (Christov et al., 2007). In return, satellite cells produce endothelial growth factors to stimulate angiogenesis and provide the new tissue with oxygen and nutrients (Dallabrida et al., 2005; Rhoads et al., 2009; Flann et al., 2014). It is likely that each of these individual growth factors plays an important role in directing the cellular responses of the local environment to ischemia. In this context, altered growth factor expression would be regulated in a temporal manner to direct muscle progenitor cell responses, i.e., proliferation and differentiation. Of particular relevance to the ischemic response are the decrease in HGF expression by muscle progenitor cells (Flann et al., 2014) and the increase in VEGF expression by progenitor cells and endothelial cells induced by hypoxia. HGF is known to mediate satellite cell quiescence in a concentration-dependent fashion (Yamada et al., 2010). In contrast, VEGF destabilizes the endothelium and induces proliferation of both endothelial cells and satellite cells. Reducing inhibitory stimuli while increasing proliferative stimuli would be an evolutionarily advantageous means of ensuring proper progenitor cell numbers to repair the myopathy during ischemia while simultaneously providing an angiogenic stimulus to restore proper delivery of oxygen and nutrients. Here, and in our recent work *in vivo* (Mofarrahi et al., 2015), we demonstrate that the Angiopoietin signaling axis is an important component of the differentiation response during skeletal muscle tissue repair. In the context of the overall signaling network between muscle cells and endothelial cells, Ang-1 likely represents a “maturation signal,” driving the maturation of both nascent vascular sprouts (Fagiani and Christofori, 2013; Qin et al., 2013) and the fusing myoblasts involved in myofiber restoration. Thus, in addition to HGF, VEGF, and possibly other growth factors, Ang-1 represents another critical component of the microenvironmental response that drives both vascular- and muscle-specific responses to ischemia.

It is important to note that Ang-1 may have differential effects on muscle and endothelial cells in both a concentration- and context-dependent manner (Dallabrida et al., 2005; Abou-Khalil et al., 2009; Fukuhara et al., 2010; Lee et al., 2013). Angiopoietin/Tie receptor signaling in the endothelium is known to be context-dependent (Eklund and Olsen, 2006), thus it is possible that this ligand-receptor system could have distinct effects on specific populations of muscle precursor cells (i.e., quiescent, proliferating, or differentiating cells). Therefore examining effects at different cellular developmental stages is important to elucidate the role of Ang-1 signaling in skeletal muscle in general and regenerating muscle specifically. In the current study, MC CM recapitulated the effects of recombinant Ang-1 on myotube formation, and this effect was abrogated by depleting Ang-1 with soluble Tie2, indicating that Ang-1 secreted by ischemic muscle fibers likely plays an important role in promoting muscle regeneration in the context of ischemic myopathy. EC CM and VEGF promoted myoblast and EC proliferation, but the decrease in VEGFRs in myoblasts during differentiation is consistent with a unique temporal regulation of effects this vascular growth factor on each cell type. Although we previously demonstrated that Ang-1 can promote skeletal muscle regeneration after cardiotoxin-mediated injury, the ischemic environment may induce unique and differential responses of endothelial and muscle cells as well as unique interactions between them. In this regard, our current results demonstrate an important temporal regulation of endothelial and skeletal muscle cells' responses to ischemia, specifically with respect to the expression of VEGF, Ang-1, and their cognate receptors. The ability of both ECs and muscle cells to proliferate in response to VEGF, which is upregulated by tissue ischemia and subsequent cellular hypoxia, facilitates the regeneration of both cellular compartments. The up regulation of Ang-1 and the Tie receptors coincides with a decrease in VEGF/VEGFR expression and remodeling of both muscle cells (i.e., fusion of myoblasts into myofibers) and endothelial cells (capillary morphogenesis and vascular maturation) during the later phases of muscle tissue regeneration. These findings provide important insights into the mechanisms by which “vascular” growth factors coordinately regulate both muscle and endothelial cell responses and they have implications for the treatment of ischemic vascular diseases.

Recent pre-clinical evidence supports the possibility that genetic differences play a key role in individuals' susceptibility to PAD, offering an opportunity to gain mechanistic insights and to develop novel interventions for this disease. Specifically, different inbred mouse strains have dramatically different responses to hind limb ischemia (HLI), a model of PAD. C57BL/6 (BL6) mice have greater baseline collateral artery density, better limb perfusion after HLI, and marked resistance to tissue necrosis. In contrast, BALB/c mice suffer appreciable tissue loss after HLI (Helisch et al., 2006; Dokun et al., 2008), which has been assumed to be a primary vascular effect. We previously established a strain dependent and muscle progenitor specific deficit in the BALB/c ischemic response *in vitro*, which included variations in the expression of vascular growth factors and their receptors (McClung et al., 2012). The current study did not identify strain specific differences in the secretion or biological activity of Ang-1 after ischemia in myotubes *in vitro*. Our previous work detailed that the exacerbated myopathy in the muscle tissue of the genetically susceptible BALB/c mice is accompanied by significant temporal disruptions in the cellular secretion of vascular growth factors such as VEGF and Ang-1(McClung et al., 2012). Temporal disruptions in growth factor release or endogenous cell activity are known to lead to progressive exacerbation of muscle tissue degeneration and fibrosis in models of muscle insult (Sato et al., 2003; Li et al., 2004; Zhu et al., 2007). The timing of cognate receptor expression in individual cell types complicates this scenario, and is also disrupted in response to an ischemic myopathy *in vivo* and experimental ischemia *in vitro* (McClung et al., 2012). In the current study, we established the ability of myotubes from both genetically susceptible (BALB/c) and protected (BL6) parental strains to secrete and/or respond to Ang-1 in a stable and carefully controlled *in vitro* environment. Further work is needed to specifically examine whether temporal alterations in the secretion of vascular growth factors or disruptions in the context dependent expression of their cognate receptors in the ischemic *in vivo* microenvironment is sufficient to drive the strain dependent myopathy.

A major weakness of prior progenitor cell therapies for PAD is an incomplete understanding of the effects of ischemia on the different cell types in skeletal muscle, including endothelial, skeletal muscle, and progenitor cells. Given the recent demonstration of muscle as an endocrine organ (Pedersen, 2011a,b) with the ability to induce angiogenic transcriptional programs and respond to angiogenic factors (McClung et al., 2012), regenerating skeletal muscle may represent a unique source of both paracrine and autocrine factors in the local ischemic environment of PAD (Renault et al., 2013). Systems of cross talk between various cell types during periods of ischemia *in vivo* remain poorly defined, but may represent complex and novel evolutionarily conserved mechanisms for overall tissue survival and recovery from ischemic insult. This is especially true in regards to the myopathy that accompanies limb ischemia. Here, we have identified a potential role for the traditional vascular growth factor Ang-1 in ischemic muscle precursor and progenitor cell biology and established the potential autocrine/paracrine function of muscle and endothelial cells to drive an organized temporal response to ischemic muscle injury. This work reveals new roles for angiogenic factors in muscle biology that could be critical to improving therapeutic strategies for PAD.

### Conflict of interest statement

The authors declare that the research was conducted in the absence of any commercial or financial relationships that could be construed as a potential conflict of interest.

## Abbreviations

MPC: muscle progenitor cell
MC: muscle cell
EC: endothelial cell
CM: conditioned medium
HND: hypoxia and nutrient deprivation
PAD: peripheral artery disease
CLI: critical limb ischemia
VEGF: vascular endothelial growth factor
VEGFR: vascular endothelial growth factor receptor
Ang: angiopoietin
HUVEC: human umbilical vein endothelial cell.

## References

[B1] Abou-Khalil R. Le Grand F. Pallafacchina G. Valable S. Authier F. J. Rudnicki M. A.. (2009). Autocrine and paracrine angiopoietin 1/Tie-2 signaling promotes muscle satellite cell self-renewal. Cell Stem Cell 5, 298–309. 10.1016/j.stem.2009.06.001 19733541 PMC4592285

[B2] Abou-Khalil R. Mounier R. Chazaud B. (2010). Regulation of myogenic stem cell behavior by vessel cells: the “menage a trois” of satellite cells, periendothelial cells and endothelial cells. Cell Cycle 9, 892–896. 10.4161/cc.9.5.10851 20160472

[B3] Arany Z. Foo S. Y. Ma Y. Ruas J. L. Bommi-Reddy A. Girnun G.. (2008). HIF-independent regulation of VEGF and angiogenesis by the transcriptional coactivator PGC-1alpha. Nature 451, 1008–1012. 10.1038/nature06613 18288196

[B4] Arsic N. Zacchigna S. Zentilin L. Ramirez-Correa G. Pattarini L. Salvi A.. (2004). Vascular endothelial growth factor stimulates skeletal muscle regeneration *in vivo*. Mol. Ther. 10, 844–854. 10.1016/j.ymthe.2004.08.007 15509502

[B5] Blum A. Balkan W. Hare J. M. (2012). Advances in cell-based therapy for peripheral vascular disease. Atherosclerosis 223, 269–277. 10.1016/j.atherosclerosis.2012.03.017 22494624

[B6] Brass E. P. Hiatt W. R. (2000). Acquired skeletal muscle metabolic myopathy in atherosclerotic peripheral arterial disease. Vasc. Med. 5, 55–59. 10.1177/1358836X0000500109 10737157

[B7] Brass E. P. Hiatt W. R. Green S. (2004). Skeletal muscle metabolic changes in peripheral arterial disease contribute to exercise intolerance: a point-counterpoint discussion. Vasc. Med. 9, 293–301. 10.1191/1358863x04vm572ra 15678622

[B8] Brass E. P. Wang H. Hiatt W. R. (2000). Multiple skeletal muscle mitochondrial DNA deletions in patients with unilateral peripheral arterial disease. Vasc. Med. 5, 225–230. 10.1177/1358836X0000500405 11213234

[B9] Charge S. B. Rudnicki M. A. (2004). Cellular and molecular regulation of muscle regeneration. Physiol. Rev. 84, 209–238. 10.1152/physrev.00019.2003 14715915

[B10] Chazaud B. Sonnet C. Lafuste P. Bassez G. Rimaniol A. C. Poron F.. (2003). Satellite cells attract monocytes and use macrophages as a support to escape apoptosis and enhance muscle growth. J. Cell Biol. 163, 1133–1143. 10.1083/jcb.200212046 14662751 PMC2173611

[B11] Cho C. H. Kammerer R. A. Lee H. J. Steinmetz M. O. Ryu Y. S. Lee S. H.. (2004). COMP-Ang1: a designed angiopoietin-1 variant with nonleaky angiogenic activity. Proc. Natl. Acad. Sci. U.S.A. 101, 5547–5552. 10.1073/pnas.0307574101 15060279 PMC397420

[B12] Christov C. Chretien F. Abou-Khalil R. Bassez G. Vallet G. Authier F. J.. (2007). Muscle satellite cells and endothelial cells: close neighbors and privileged partners. Mol. Biol. Cell 18, 1397–1409. 10.1091/mbc.E06-08-0693 17287398 PMC1838982

[B13] Dallabrida S. M. Ismail N. Oberle J. R. Himes B. E. Rupnick M. A. (2005). Angiopoietin-1 promotes cardiac and skeletal myocyte survival through integrins. Circ. Res. 96, e8–e24. 10.1161/01.RES.0000158285.57191.60 15692086

[B14] Davis S. Aldrich T. H. Jones P. F. Acheson A. Compton D. L. Jain V.. (1996). Isolation of angiopoietin-1, a ligand for the TIE2 receptor, by secretion-trap expression cloning. Cell 87, 1161–1169. 10.1016/S0092-8674(00)81812-7 8980223

[B15] Deasy B. M. Feduska J. M. Payne T. R. Li Y. Ambrosio F. Huard J. (2009). Effect of VEGF on the regenerative capacity of muscle stem cells in dystrophic skeletal muscle. Mol. Ther. 17, 1788–1798. 10.1038/mt.2009.136 19603004 PMC2835014

[B16] Dokun A. O. Keum S. Hazarika S. Li Y. Lamonte G. M. Wheeler F.. (2008). A quantitative trait locus (LSq-1) on mouse chromosome 7 is linked to the absence of tissue loss after surgical hindlimb ischemia. Circulation 117, 1207–1215. 10.1161/CIRCULATIONAHA.107.736447 18285563 PMC2881228

[B17] Eklund L. Olsen B. R. (2006). Tie receptors and their angiopoietin ligands are context-dependent regulators of vascular remodeling. Exp. Cell Res. 312, 630–641. 10.1016/j.yexcr.2005.09.002 16225862

[B18] Fadini G. P. Agostini C. Avogaro A. (2010). Autologous stem cell therapy for peripheral arterial disease meta-analysis and systematic review of the literature. Atherosclerosis 209, 10–17. 10.1016/j.atherosclerosis.2009.08.033 19740466

[B19] Fadini G. P. Losordo D. Dimmeler S. (2012). Critical reevaluation of endothelial progenitor cell phenotypes for therapeutic and diagnostic use. Circ. Res. 110, 624–637. 10.1161/CIRCRESAHA.111.243386 22343557 PMC3382070

[B20] Fagiani E. Christofori G. (2013). Angiopoietins in angiogenesis. Cancer Lett. 328, 18–26. 10.1016/j.canlet.2012.08.018 22922303

[B21] Flann K. L. Rathbone C. R. Cole L. C. Liu X. Allen R. E. Rhoads R. P. (2014). Hypoxia simultaneously alters satellite cell-mediated angiogenesis and hepatocyte growth factor expression. J. Cell. Physiol. 229, 572–579. 10.1002/jcp.24479 24122166

[B22] Fukuhara S. Sako K. Noda K. Zhang J. Minami M. Mochizuki N. (2010). Angiopoietin-1/Tie2 receptor signaling in vascular quiescence and angiogenesis. Histol. Histopathol. 25, 387–396. 20054809 10.14670/HH-25.387

[B23] Gehrig S. M. Lynch G. S. (2011). Emerging drugs for treating skeletal muscle injury and promoting muscle repair. Expert Opin. Emerg. Drugs 16, 163–182. 10.1517/14728214.2010.524743 21323590

[B24] Germani A. Di Carlo A. Mangoni A. Straino S. Giacinti C. Turrini P.. (2003). Vascular endothelial growth factor modulates skeletal myoblast function. Am. J. Pathol. 163, 1417–1428. 10.1016/S0002-9440(10)63499-2 14507649 PMC1868307

[B25] Harfouche R. Gratton J. P. Yancopoulos G. D. Noseda M. Karsan A. Hussain S. N. (2003). Angiopoietin-1 activates both anti- and proapoptotic mitogen-activated protein kinases. FASEB J. 17, 1523–1525. 10.1096/fj.02-0698fje 12824293

[B26] Helisch A. Wagner S. Khan N. Drinane M. Wolfram S. Heil M.. (2006). Impact of mouse strain differences in innate hindlimb collateral vasculature. Arterioscler. Thromb. Vasc. Biol. 26, 520–526. 10.1161/01.ATV.0000202677.55012.a0 16397137

[B27] Hirsch A. T. Haskal Z. J. Hertzer N. R. Bakal C. W. Creager M. A. Halperin J. L.. (2006). ACC/AHA 2005 Practice Guidelines for the management of patients with peripheral arterial disease (lower extremity, renal, mesenteric, and abdominal aortic): a collaborative report from the American Association for Vascular Surgery/Society for Vascular Surgery, Society for Cardiovascular Angiography and Interventions, Society for Vascular Medicine and Biology, Society of Interventional Radiology, and the ACC/AHA Task Force on Practice Guidelines (Writing Committee to Develop Guidelines for the Management of Patients With Peripheral Arterial Disease): endorsed by the American Association of Cardiovascular and Pulmonary Rehabilitation; National Heart, Lung, and Blood Institute; Society for Vascular Nursing; TransAtlantic Inter-Society Consensus; and Vascular Disease Foundation. Circulation 113, e463–e654. 10.1161/CIRCULATIONAHA.106.174526 16549646

[B28] Iwaguro H. Yamaguchi J. Kalka C. Murasawa S. Masuda H. Hayashi S.. (2002). Endothelial progenitor cell vascular endothelial growth factor gene transfer for vascular regeneration. Circulation 105, 732–738. 10.1161/hc0602.103673 11839630

[B29] Jazwa A. Tomczyk M. Taha H. M. Hytonen E. Stoszko M. Zentilin L.. (2013). Arteriogenic therapy based on simultaneous delivery of VEGF-A and FGF4 genes improves the recovery from acute limb ischemia. Vasc. Cell 5:13. 10.1186/2045-824X-5-13 23816205 PMC3703285

[B30] Karkkainen A. M. Kotimaa A. Huusko J. Kholova I. Heinonen S. E. Stefanska A.. (2009). Vascular endothelial growth factor-D transgenic mice show enhanced blood capillary density, improved postischemic muscle regeneration, and increased susceptibility to tumor formation. Blood 113, 4468–4475. 10.1182/blood-2008-07-171108 19074006

[B31] Lawall H. Bramlage P. Amann B. (2011). Treatment of peripheral arterial disease using stem and progenitor cell therapy. J. Vasc. Surg. 53, 445–453. 10.1016/j.jvs.2010.08.060 21030198

[B32] Lee E. H. Woo J. S. Hwang J. H. Park J. H. Cho C. H. (2013). Angiopoietin 1 enhances the proliferation and differentiation of skeletal myoblasts. J. Cell. Physiol. 228, 1038–1044. 10.1002/jcp.24251 23041942

[B33] Li Y. Foster W. Deasy B. M. Chan Y. Prisk V. Tang Y.. (2004). Transforming growth factor-beta1 induces the differentiation of myogenic cells into fibrotic cells in injured skeletal muscle: a key event in muscle fibrogenesis. Am. J. Pathol. 164, 1007–1019. 10.1016/S0002-9440(10)63188-4 14982854 PMC1614716

[B34] Lin P. Buxton J. A. Acheson A. Radziejewski C. Maisonpierre P. C. Yancopoulos G. D.. (1998). Antiangiogenic gene therapy targeting the endothelium-specific receptor tyrosine kinase Tie2. Proc. Natl. Acad. Sci. U.S.A. 95, 8829–8834. 10.1073/pnas.95.15.8829 9671764 PMC21162

[B35] McClung J. M. Mccord T. J. Keum S. Johnson S. Annex B. H. Marchuk D. A.. (2012). Skeletal muscle-specific genetic determinants contribute to the differential strain-dependent effects of hindlimb ischemia in mice. Am. J. Pathol. 180, 2156–2169. 10.1016/j.ajpath.2012.01.032 22445571 PMC3349830

[B36] McDermott M. M. Liu K. Tian L. Guralnik J. M. Criqui M. H. Liao Y.. (2012). Calf muscle characteristics, strength measures, and mortality in peripheral arterial disease: a longitudinal study. J. Am. Coll. Cardiol. 59, 1159–1167. 10.1016/j.jacc.2011.12.019 22440216 PMC3465664

[B37] Messina S. Mazzeo A. Bitto A. Aguennouz M. Migliorato A. De Pasquale M. G.. (2007). VEGF overexpression via adeno-associated virus gene transfer promotes skeletal muscle regeneration and enhances muscle function in mdx mice. FASEB J. 21, 3737–3746. 10.1096/fj.07-8459com 17575261

[B38] Misteli H. Wolff T. Fuglistaler P. Gianni-Barrera R. Gurke L. Heberer M.. (2010). High-throughput flow cytometry purification of transduced progenitors expressing defined levels of vascular endothelial growth factor induces controlled angiogenesis *in vivo*. Stem cells 28, 611–619. 10.1002/stem.291 20039367

[B39] Mofarrahi M. Mcclung J. M. Kontos C. D. Davis E. C. Tappuni B. Moroz N.. (2015). Angiopoietin-1 Enhances Skeletal Muscle Regeneration in Mice. Am. J. Physiol. Regul. Integr. Comp. Physiol. 308, R576–R589. 10.1152/ajpregu.00267.2014 25608750 PMC4386004

[B40] Pedersen B. K. (2011a). Exercise-induced myokines and their role in chronic diseases. Brain Behav. Immun. 25, 811–816. 10.1016/j.bbi.2011.02.010 21354469

[B41] Pedersen B. K. (2011b). Muscles and their myokines. J. Exp. Biol. 214, 337–346. 10.1242/jeb.048074 21177953

[B42] Pipinos I. I. Judge A. R. Selsby J. T. Zhu Z. Swanson S. A. Nella A. A.. (2007). The myopathy of peripheral arterial occlusive disease: part 1. Functional and histomorphological changes and evidence for mitochondrial dysfunction. Vasc. Endovascular Surg. 41, 481–489. 10.1177/1538574407311106 18166628

[B43] Pipinos I. I. Judge A. R. Selsby J. T. Zhu Z. Swanson S. A. Nella A. A.. (2008). The myopathy of peripheral arterial occlusive disease: part 2. Oxidative stress, neuropathy, and shift in muscle fiber type. Vasc. Endovascular Surg. 42, 101–112. 10.1177/1538574408315995 18390972 PMC12282609

[B44] Prather W. R. Toren A. Meiron M. Ofir R. Tschope C. Horwitz E. M. (2009). The role of placental-derived adherent stromal cell (PLX-PAD) in the treatment of critical limb ischemia. Cytotherapy 11, 427–434. 10.1080/14653240902849762 19526389

[B45] Qin D. Trenkwalder T. Lee S. Chillo O. Deindl E. Kupatt C.. (2013). Early vessel destabilization mediated by Angiopoietin-2 and subsequent vessel maturation via Angiopoietin-1 induce functional neovasculature after ischemia. PLoS ONE 8:e61831. 10.1371/journal.pone.0061831 23613948 PMC3628915

[B46] Reiss Y. (2010). Angiopoietins. Recent Results Cancer Res. 180, 3–13. 10.1007/978-3-540-78281-0_2 20033375

[B47] Renault M. A. Chapouly C. Yao Q. Larrieu-Lahargue F. Vandierdonck S. Reynaud A.. (2013). Desert hedgehog promotes ischemia-induced angiogenesis by ensuring peripheral nerve survival. Circ. Res. 112, 762–770. 10.1161/CIRCRESAHA.113.300871 23343527

[B48] Rhoads R. P. Johnson R. M. Rathbone C. R. Liu X. Temm-Grove C. Sheehan S. M.. (2009). Satellite cell-mediated angiogenesis *in vitro* coincides with a functional hypoxia-inducible factor pathway. Am. J. Physiol. Cell Physiol. 296, C1321–C1328. 10.1152/ajpcell.00391.2008 19386789 PMC2692418

[B49] Rissanen T. T. Vajanto I. Hiltunen M. O. Rutanen J. Kettunen M. I. Niemi M.. (2002). Expression of vascular endothelial growth factor and vascular endothelial growth factor receptor-2 (KDR/Flk-1) in ischemic skeletal muscle and its regeneration. Am. J. Pathol. 160, 1393–1403. 10.1016/S0002-9440(10)62566-7 11943724 PMC1867222

[B50] Rissanen T. T. Vajanto I. Yla-Herttuala S. (2001). Gene therapy for therapeutic angiogenesis in critically ischaemic lower limb - on the way to the clinic. Eur. J. Clin. Invest. 31, 651–666. 10.1046/j.1365-2362.2001.00864.x 11473566

[B51] Rosenblatt J. D. Lunt A. I. Parry D. J. Partridge T. A. (1995). Culturing satellite cells from living single muscle fiber explants. In Vitro Cell. Dev. Biol. Anim. 31, 773–779. 10.1007/BF02634119 8564066

[B52] Sato K. Li Y. Foster W. Fukushima K. Badlani N. Adachi N.. (2003). Improvement of muscle healing through enhancement of muscle regeneration and prevention of fibrosis. Muscle Nerve 28, 365–372. 10.1002/mus.10436 12929198

[B53] Volz K. S. Miljan E. Khoo A. Cooke J. P. (2012). Development of pluripotent stem cells for vascular therapy. Vascul. Pharmacol. 56, 288–296. 10.1016/j.vph.2012.02.010 22387745 PMC3595194

[B54] Von Degenfeld G. Banfi A. Springer M. L. Blau H. M. (2003). Myoblast-mediated gene transfer for therapeutic angiogenesis and arteriogenesis. Br. J. Pharmacol. 140, 620–626. 10.1038/sj.bjp.0705492 14534145 PMC1574078

[B55] Wagatsuma A. (2007). Endogenous expression of angiogenesis-related factors in response to muscle injury. Mol. Cell. Biochem. 298, 151–159. 10.1007/s11010-006-9361-x 17435971

[B56] Winkler T. Von Roth P. Matziolis G. Schumann M. R. Hahn S. Strube P.. (2011). Time course of skeletal muscle regeneration after severe trauma. Acta Orthop. 82, 102–111. 10.3109/17453674.2010.539498 21142822 PMC3230005

[B57] Yamada M. Tatsumi R. Yamanouchi K. Hosoyama T. Shiratsuchi S. Sato A.. (2010). High concentrations of HGF inhibit skeletal muscle satellite cell proliferation *in vitro* by inducing expression of myostatin: a possible mechanism for reestablishing satellite cell quiescence *in vivo*. Am. J. Physiol. Cell Physiol. 298, C465–C476. 10.1152/ajpcell.00449.2009 20007454 PMC2838568

[B58] Yan H. Guo Y. Zhang P. Zu L. Dong X. Chen L.. (2005). Superior neovascularization and muscle regeneration in ischemic skeletal muscles following VEGF gene transfer by rAAV1 pseudotyped vectors. Biochem. Biophys. Res. Commun. 336, 287–298. 10.1016/j.bbrc.2005.08.066 16129416

[B59] Yang Y. Tang G. Yan J. Park B. Hoffman A. Tie G.. (2008). Cellular and molecular mechanism regulating blood flow recovery in acute versus gradual femoral artery occlusion are distinct in the mouse. J. Vasc. Surg. 48, 1546–1558. 10.1016/j.jvs.2008.07.063 19118738 PMC2791875

[B60] Zhu J. Li Y. Shen W. Qiao C. Ambrosio F. Lavasani M.. (2007). Relationships between transforming growth factor-beta1, myostatin, and decorin: implications for skeletal muscle fibrosis. J. Biol. Chem. 282, 25852–25863. 10.1074/jbc.M704146200 17597062

